# Integration of gene expression and DNA-methylation profiles improves molecular subtype classification in acute myeloid leukemia

**DOI:** 10.1186/1471-2105-16-S4-S5

**Published:** 2015-02-23

**Authors:** Erdogan Taskesen, Sepideh Babaei, Marcel MJ Reinders, Jeroen de Ridder

**Affiliations:** 1 Delft Bioinformatics Lab (DBL), Delft, Netherlands; 2 Netherlands Bioinformatics Centre (NBIC), Netherlands

**Keywords:** Acute Myeloid Leukemia, gene expression profiles, DNA-methylation profiles, AML subtypes classification

## Abstract

**Background:**

Acute Myeloid Leukemia (AML) is characterized by various cytogenetic and molecular abnormalities. Detection of these abnormalities is important in the risk-classification of patients but requires laborious experimentation. Various studies showed that gene expression profiles (GEP), and the gene signatures derived from GEP, can be used for the prediction of subtypes in AML. Similarly, successful prediction was also achieved by exploiting DNA-methylation profiles (DMP). There are, however, no studies that compared classification accuracy and performance between GEP and DMP, neither are there studies that integrated both types of data to determine whether predictive power can be improved.

**Approach:**

Here, we used 344 well-characterized AML samples for which both gene expression and DNA-methylation profiles are available. We created three different classification strategies including early, late and no integration of these datasets and used them to predict AML subtypes using a logistic regression model with Lasso regularization.

**Results:**

We illustrate that both gene expression and DNA-methylation profiles contain distinct patterns that contribute to discriminating AML subtypes and that an integration strategy can exploit these patterns to achieve synergy between both data types. We show that concatenation of features from both data sets, i.e. early integration, improves the predictive power compared to classifiers trained on GEP or DMP alone. A more sophisticated strategy, i.e. the late integration strategy, employs a two-layer classifier which outperforms the early integration strategy.

**Conclusion:**

We demonstrate that prediction of known cytogenetic and molecular abnormalities in AML can be further improved by integrating GEP and DMP profiles.

## Introduction

Over the last decade, microarray technologies that measures gene expression profiles (GEP) proved to be effective for the detection of biomarkers for diagnosis and prognosis of disease and for helping with the determination of drug treatment [1]. More recently, microarray-based GEP is emerging as a predictive tool to further refine risk stratification in patients with myeloma [2]. In this study, we focused on patients with Acute Myeloid Leukemia (AML). AML is a heterogeneous disease for which groups of patients can be identified with common cytogenetic or molecular abnormality (denoted as subtypes) [3]. Many abnormalities in AML, such as in gene fms-like tyro-sine kinase 3 (*FLT3*), nucleophosmin (*NPM1 *) or CCAAT enhancer binding protein alpha (*CEBPA*), are nowadays used for risk-classification of patients [4,5]. Currently, laborious diagnostic techniques are used to detect these cytogenetical or molecular abnormalities, such as direct Sanger sequencing on patients with an abnormal denaturing high-performance liquid chromatography profile (dHPLC) [6]. Alternatively, AML subtypes can also be determined based on predictive gene signatures derived using classification models from a training dataset with known subtypes. Therefore, a better classification performance and robust gene signature is highly relevant for future clinical practice. Although it has already been demonstrated that some subtypes can be predicted very well using GEP, e.g. t(8;21), t(15;17), inv(16), or *CEBPA^double-mutant ^*[7], challenges lie ahead for subtypes such as patients carrying abnormalities in *FLT3*, *NPM1*, or with certain chromosomal abnormalities (3q, 11q23, 5q, 7q) [8], where it is much more difficult to predict these accurately. This is an indication that gene expression profiles do not contain features that are sufficiently discriminative to distinguish those groups of patients from the others.

Besides gene expression profiles, DNA-methylation profiles (DMP) also provide insight into the pathology of Acute Myeloid Leukemia (AML) [9]. In particular t(8;21), t(15;17), and inv(16) leukemia entities are associated with specific methylation profiles, and four epigenetic distinct forms of AML with *NPM1 *mutations are detected [9]. This suggests that a number of genes may be regulated at the methylation level and consequently could be included in predictive gene signatures. Another example is CCAAT-enhancer binding protein alpha *CEBPA*, which is an important transcription factor in AML and a mutation (e.g. double mutation in *CEBPA*) can cause a selective block in differentiation, a hallmark of AML [10,11]. However, there are patients that are highly similar to the *CEBPA^double-mutant ^*cases, but do not harbour this mutation. Follow-up experimentation showed that these patients contained a unique epigenetic feature, i.e. *CEBPA *is silenced by DNA hypermethylation (denoted as the *CEBPA^silenced ^*subtype) [12]. Thus, by only looking at mRNA expression levels, similar expression patterns for different phenotypes can arise. However, if epigenetic regulation is incorporated, e.g. the promoter methylation of genes is taken into account while deriving predictive gene signatures, such underlying patterns may be disclosed.

The combination of GEP with other data types measured in the same sample is not frequently available. To derive more reliable and robust gene signatures and improve classification performance many methods have therefore been developed that integrate GEP with prior knowledge. Some examples include integration with protein-protein networks [13,14], protein sequences similarities [15], pathways, gene ontology or other functional groups of genes [16]. Although such additional data can approximate the functional roles of genes, it is more powerful to include additional measurements on the same set of samples to directly probe the relevance of biological processes within these samples. This is demonstrated in studies where multiple data sets are integrated to improve classification power, such as integration of GEP with clinical data [17], GEP with single-nucleotide polymorphisms [18], or GEP with Copy Number Variations and other clinical information [19].

We hypothesize that the prediction of AML subtypes can be improved by combining gene expression and DNA-methylation profiles, as it has previously been shown that DNA-methytlation patterns contain complementary information on top of the gene expression patterns in an unsupervised analysis. For example, it has been shown that integrating GEP and DMP revealed two unique, and clinically relevant AML patient-clusters that could not be discovered when using either GEP or DMP alone [20].

While both gene expression and DNA-methylation profiles have been used to gain insight in AML [3,9], there are, to our knowledge, currently no studies that describe how they should be integrated to exploit possible synergies between these data types for prediction purposes. In fact there are many ways in which multiple measurements in the same sample can be integrated. In this study we propose and investigate two integration strategies, early and late integration, and compare the classification performance with that obtained using the two data types separately, i.e. no integration.

## Results

### Gene expression profiles outperform DNA-methylation profiles

**Figure 1:**
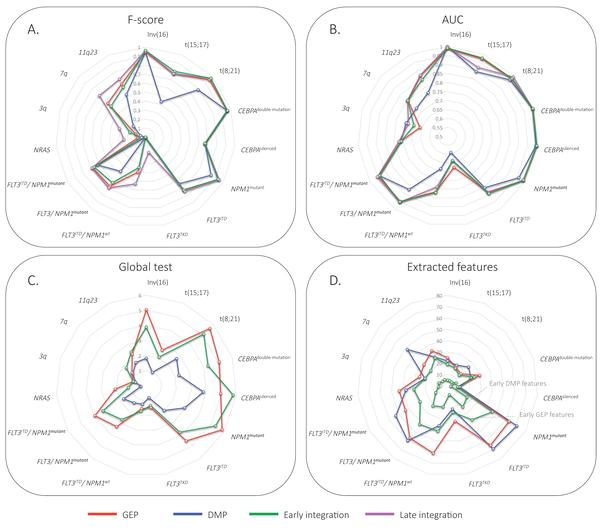
**Classification performance for different integration strategies**. A) Classification accuracy based on the DLCV scheme (F-score), B) Classification performance based on the DLCV scheme (AUC). C) Resulting -log10(*P*-values) of global test based on the training and test sets. D) Number of features extracted by logistic regression model from training subsets. Note that the late integration is based on the extracted features from solely GEP and DMP.

We compared the predictive power for AML subtypes by independently assessing the predictive power of GEP and DMP. We found that the use of mRNA expression profiles equals or outperforms the DNA-methylation profiles in classification accuracy (F-score, Figure 1A andAdditional file 1: Table S1) for all subtypes. The best results for DMP are obtained for the subtypes, inv(16), *CEBPA^double-mutation^*, and *CEBPA^silenced^*. However, the accuracy (F-score, 0.9455, 0.9556, and 0.6667 respectively) and classification performance (AUC, 0.9995, 1, and 0.9955 respectively, Figure 1B andAdditional file 1: Table S1) is similar to those obtained using GEP. Thus, in terms of predictive power (accuracy or performance) it does not matter whether GEP or DMP is used to classify the latter three subtypes. On the other hand, the results obtained with the global test clearly show that features from GEP are more significantly associated with the subtypes than features from DMP. For instance, inv(16) (*P_GEP _*= 8.95*E *- 06, *P_DMP _*= 0.014), *CEBPA^double-mutation ^*(*P_GEP _*= 8.21*E *- 06, *P_DMP _*= 0.0053), and *CEBPA^silenced ^*(*P_GEP _*= 1.11*E *- 05, *P_DMP _*= 1.5*E *- 04). This trend is also apparent for the other subtypes (Figure 1C andAdditional file 1: Table S1). Although the AUC shows minimal difference between GEP and DMP over all AML subtypes, the GEP features are more discriminative.

### Early integration improves (or equals) predictive power

The early integration strategy improves (or equals) the classification accuracy for all 15 subtypes compared to DMP, and for 8 out of 15 subtypes for GEP (Figure 1A andAdditional file 1: Table S1). On the other hand, the performance improves (or equals) for 10 out of 15 subtypes compared to GEP and 12 out of 15 subtypes for DMP alone. This demonstrates that integrating methylation and expression profiles is useful for the prediction of the leukemia subtypes. This is especially the case for the recently identified *CEBPA^silenced ^*subtype for which it is known that it contains a unique epigenetic signature [12]. The global test revealed that the use of the combined feature space results in increased associations for this subtype *P_EARLY _*= 1.6*E *- 06, compared to features selected based on GEP alone (*P_GEP _*= 1.0*E *- 05) or DMP alone (*P_DMP _*= 1.5*E *- 04). Nevertheless, the classification accuracy was equal for all integration strategies (*F *- *score *= 0.667).

In terms of number of features selected by the classifier, we can read from Figure 1D (and Additional file 1: Table S1) that a similar number is selected when trained on the GEP and DMP separately. Moreover, the proportions between GEP and DMP features are approximately the same over the subtypes (*r *= 0.91). Interestingly, the selected features obtained with the early integration strategy are composed of both GEP and DMP features (Figure 1D, green line). Strikingly, their proportions are also similar (*r *= 0.92).

To summarize, an early integration strategy can improve subtype classification. However, for some subtypes the classification performance is equal to that obtained using GEP or DMP alone. The latter observation may be explained because no complementary information is carried in the DNA-methylation and gene expression profiles for certain subtypes. If this is the case, early integration may even result in loss of performance as the dimensionality of the original feature space approximately doubles in size. As a result, classification will suffer more from the small sample size problem.

### Late integration demonstrates best classification accuracy

**Figure 2:**
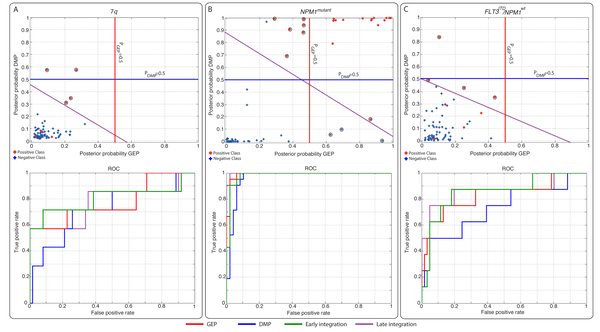
**Classification results on the validation sets and their corresponding ROC curves**. Top illustrates scatter plots of the logistic regression classifier (first layer) outcomes on a validation subset for subtype (A) 7q (subset5), (B) *NPM1^mutant ^*(subset2), and (C) *FLT3^ITD^*/*NPM1^wt ^*(subset3). Red and blue solid lines indicate the classification boundaries of the first layer (GEP and DMP respectively). The purple solid line indicates the classification boundary of the second layer. Black encapsulated circles indicates samples that are misclassified when we trained the first layer on GEP or DMP only. These are however correctly classified when we incorporate the the second layer. Bottom illustrates the ROC curves of the scatter plots.

The best subtype classification accuracy was obtained by a late integration strategy. It outperformed GEP, DMP and early integration for all AML subtypes with one exception; patients with t(15;17) showed better accuracy in the early integration (*F - score *= 0.8) compared to the late integration (*F - score *= 0.77). The better accuracy of the late integration can be explained in two ways: first, in the early integration strategy we standardized all features to the same scale to make them equally important, whereas in the late integration no additional normalization is required for GEP and DMP as we train the 2nd layer classifier on the outputs of the GEP and DMP regressors. Secondly, the threshold on the posterior probability is not fixed in the late integration scheme but learned by the nearest mean classifier (NMC). The latter is illustrated in Figure 2, where circles illustrate samples for which the late integration makes a difference between correct (in late integration) or wrong (in separate) classification. A clear example is given by the set of 7q patients (Figure 2A) that improved in the classification accuracy and performance (*AUC *= 0.799, *F - score *= 0.68) compared to GEP (*AUC *= 0.794, *F - score *= 0.56), DMP (*AUC *= 0.733, *F - score *= 0.18) individually or early integration (*AUC *= 0.799, *F - score *= 0.51) (Figure 1A and 1B). Other sets of samples with a marked difference are the *NPM1*^*mutant *^and *FLT3*^*ITD*^/*NPM1*^*wt *^patients as shown in Figure 2B and 2C respectively.

Interestingly, the late integration approach is also effective in cases where one of the data types was not significantly associated with the subtype, such as for cases with *FLT3^ITD^*/*NPM1^wt ^*(*P*_*GEP *_= 0.0284 and *P*_*DMP *_= 0.057, Figure 1C). In this case the classification accuracy was increased from 0.39 by using GEP to 0.52 using late integration (Figure 1A and 2C).

## Conclusions

In this study we demonstrate that integration of gene expression and DNAmethylation profiles can improve the classification performance. We show this by using an AML dataset that contains 15 well-characterized cytogenetical and molecular subtypes. These results show that both data types are synergistic, meaning that complementary patterns are present in both data types that lead to a better prediction of the leukemia subtypes. Although we demonstrate improved predictive power by using early and late integration, some subtypes only showed small improvements, such as for the AML patients with favourable risk, i.e. t(8;21), t(15;17) or inv(16). Nevertheless, the global test showed in these subtypes that the features in an early integration strategy were more indicative for the subtype compared to DMP, underpinning the observation that gene expression and methylation are complementary measurements when subtyping leukemia patients.

Patients with *NRAS*, *FLT3^TKD^*, 3q, 7q and 11q23 abnormalities showed improved prediction using the late integration strategy but nevertheless, remain difficult to predict and therefore still require alternative methods. This may be due to the high variability of expression or methylation profiles within the subtype, consequently features based on GEP or DMP cannot accurately characterize the subtype. In fact, the test statistic from the global test indeed indicates no significant association with 3q and 7q patients by using the features from either the GEP (*P *= 0.122 and *P *= 0.051 respectively) or DMP (*P *= 0.173 and *P *= 0.06 respectively). Perhaps for these cases, a multitude of other complementary data sets, such as microRNA, Copy Number Variation (CNV) or pathway information, is necessary to delineate these subtypes.

In this study we have chosen to build a classifier that is optimized for predictive power and the derived signatures may therefore not be appropriate to study the biological effect. We demonstrate a supervised approach that can improve the predictive power for each cytogenetic and molecular abnormality by employing an early or late integration strategy, whereas the late integration strategy is recommended above early integration.

## Material and methods

### AML dataset

For 344 adults, clinical, cytogenetical and molecular characteristics were analysed using bone marrow or peripheral blood, as described previously [3,9]. For each patient sample, genome-wide mRNA expression data (GEP) is measured using Affymetrix HGU133 plus2.0 (Santa Clara, CA, USA). Normalization of raw data was processed with RMA [21-23] and probes on the array are remapped to refseq transcripts using a custom chip definition file (CDF) [24] (*N_GEP _*= 21678 refseqs). For the same set of samples, genome-wide DNA-methylation data (DMP) was measured with the HELP-assay [25] and pre-processed as described previously [9] (*N_DMP _*= 22725 features). Both datasets are annotated using UCSC hg19 genome build, and are available from the NCBI Gene Expression Omnibus accession numbers GSE14468 (HOVON-SAKK cohort) and GSE18700, respectively.

### Cytogenetical and molecular abnormalities in AML

Groups of AML patients that are characterized by common cytogenetic or molecular abnormality are denoted as subtypes. We studied fifteen of the most common subtypes, which can roughly be categorized into three risk groups (good, intermediate and poor). AML subtypes in the good risk group are inv(16), t(15;17), t(8;21). The intermediate risk group contains patients with molecular abnormalities, i.e. *CEBPA^double-mutant^*, *CEBPA^silenced^*, *NPM1^mutant^*, *FLT3^ITD ^*, *FLT3^TKD^*, *FLT3^ITD^*/*NPM1^wt^*, *FLT3 */*NPM1^mutant^*, *FLT3^ITD^*/*NPM1^mutant^*, and *NRAS *cases. The poor risk group contains patients with complex karyotype (patients with more than 3 cytogenetic abnormalities), i.e. 3q, 7q, and 11q23 cases. We used the 15 subtypes as classification labels. Note that samples can contain multiple abnormalities.

### Classification strategies

**Figure 3:**
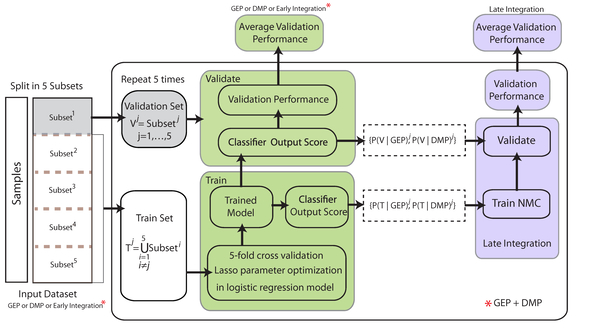
**Schematic overview of classification approach along with integration strategies**. The left part shows no and early integration strategies by training the logistic regression classifier on only GEP or DMP and GEP+DMP, respectively. In the DLCV scheme we split the input data into five subsets. The classifier was trained by means of a 5-fold cross-validation approach using four subsets for training and testing and one for validation. The right part shows the late integration procedure where the nearest mean classifier (NMC) (i.e. second layer) was trained on the new two-dimensional data which represents the first layer outcomes for GEP and DMP sets. The second layer was evaluated by the first layer outcomes of the validation subset. The reported performance is the average of classification performance on the 5 validation subsets.

The AML subtypes are classified using three different strategies: i) no integration using the GEP or DMP-dataset individually); ii) early integration; and iii) late integration (Figure 3). For each subtype, we train a two class classifier to distinguish between samples that belong to the subtype and samples that belong to the other 14 subtypes (i.e., one versus all classification scheme). We employed the logistic regression classifier with lasso regularization [26,27], which optimizes the regression output and selects features by enforcing sparsity. To assure unbiased measurements of the performance of the classifier we followed the double-loop crossvalidation protocol (DLCV) [28]. First, we split the input set into five equal subsets (the outer loop). For each iteration we use one subset as validation set and the other four subsets as input set for classifier training. Training of the classifier is based on a 5-fold cross-validation scheme (the inner loop) to optimize the regression model parameters (see Figure 3 for more details). To make sure that each feature is penalized similarly by lasso regressor, we standardized each feature to its unit second central moment before applying penalization.

#### i No integration

For the GEP and the DMP input datasets we followed the DLCV scheme, for each of the datasets separately. The optimal set of features, i.e. those discriminating between patients from one subtype and the remaining patients, were determined in the DLCV inner loop. Subsequently, we used this set of features to classify samples in the independent validation set (DLCV outer loop) and then calculate the classification performance and accuracy for each data type.

#### ii Early integration

In this strategy, we combined all the features by concatenating the GEP and DMP features yielding *N_TOTAL _*= 44403 features. Then, we followed the DLCV scheme, with the exception that we now provided the regression model with all features.

#### iii Late integration

For the late integration strategy, we established a two-layer classifier (Figure 3). Initially, we followed the DLCV scheme for each data type separately. Each inner loop generates two optimized sets of parameters for the logistic regression model, one set for each data type. In the next step we train an additional classifier, nearest mean classifier (NMC), that uses the posterior probabilities of the GEP and DMP logistic regressors as feature space. Hence, the integration of the two data types is achieved by exploiting the confidence with each individual data type. Finally the output of the NMC is evaluated on the validation set.

### Classification accuracy and performance

#### F-score

The F-score is used to test the prediction accuracy, which considers both the positive predictive value (precision) and the true positive rate (recall or sensitivity), and varies between 0 (worst accuracy) to 1 (best accuracy). To assign a sample to a class, we used the default threshold of 0.5 on the posterior probability obtained from the logistic regressor. As a result, samples for which the posterior probability is between 0.5 and 1.0 are assigned to the subtype of interest. The F-score is especially of interest in diagnostic settings where it is important to know how many patients (samples) are correctly or wrongly classified.

#### Area under the curve (AUC)

The Area under the curve (AUC) is computed on the Receiver Operator Characteristics (ROC), which integrates performance scores over all possible thresholds on the posterior probability obtained from the regressor. This effectively considers all possible assignments of samples to the subtype by the regressor.

#### Global test

To examine whether the global pattern of an input set significantly associates with the subtype, we apply the global test method [29]. The use of the global test in the evaluation is important beacuse the classification accuracy (F-score) and the classification performance (AUC) describe only the classifier output scores on the test set. The global test on the other hand results in a *P*-value based on the null hypothesis that there is no information in the given input features related to the sample label (e.g. subtype). In fact, the global test method applies a regression model to test the null hypotheses that the variance of regression coefficients of all input features is zero and subsequently calculates a test statistics.

## Competing interests

The authors declare that they have no competing interests.

## Authors' contributions

ET and SB analysed and interpreted the data, and drafted the manuscript. ET, SB, MJTR, and JdR contributed to design of the study, the discussions and editing of the manuscript. All authors provided relevant input at different stages of the project, and read and approved the final manuscript.

## Supplementary Material

Additional file 1 **Table S1**. Table containing the classification performance for different integration strategies. Click here for file

## Declarations

Publication costs for this article were sourced from the BioRange program of the Netherlands Bioinformatics Centre (NBIC), which is supported by the Netherlands Genomics Initiative (NGI, Project: BIOPATH BR 2.5). JdR is funded by the Netherlands Organisation for Scientific Research (NWO-Veni: 639.021.233).

This article has been published as part of *BMC Bioinformatics *Volume 16 Supplement 4, 2015: Selected articles from the 9th IAPR conference on Pattern Recognition in Bioinformatics. The full contents of the supplement are available online at http://www.biomedcentral.com/bmcbioinformatics/supplements/16/S4.
