# Supplementary material for: Integration of gene expression and DNA-methylation profiles improves molecular subtype classification in acute myeloid leukemia
Source: BMC Bioinformatics. 2015 Feb 23;16(Suppl 4):S5. doi: 10.1186/1471-2105-16-S4-S5 (PMC4347619; doi:10.1186/1471-2105-16-S4-S5)
Supplement: Additional file 1 — Table S1. Table containing the classification performance for different integration strategies. [file 1471-2105-16-S4-S5-S1.pdf]

Supplementary Table 1

		AUC				F-score				Global Test -log10(Pvalue)			Extracted Features			
Samples	AML_subtype	GEP	DMP	Early integration	Late integration	GEP	DMP	Early integration	Late integration	GEP	DMP	Early integration	GEP	DMP	Early integration (GEP)	Early integration (DMP)
29	**inv(16)**	0.9884	0.9995	0.9921	0.9989	0.9483	0.9455	0.9636	0.9636	5.048	1.828	3.889	25	22	19	5
12	**t(15;17)**	0.9717	0.894	0.9773	0.9197	0.7714	0.4333	0.8	0.7714	2.596	0.983	2.085	18	20	17	5
25	**t(8;21)**	0.98	0.9691	0.9825	0.9931	0.9556	0.7889	0.9778	0.9778	5.678	2.698	5.127	16	25	14	5
24	***CEBPA*** $^{double\text{-}mutation}$	0.9988	1	1	1	0.9596	0.9556	0.9596	0.9596	5.086	2.270	4.311	30	22	25	8
9	***CEBPA*** ^{silenced}	1	0.9955	1	1	0.6667	0.6667	0.6667	0.6667	4.955	3.809	5.797	9	11	9	3
105	***NPM1*** ^{mutant}	0.9888	0.9592	0.9874	0.9873	0.9353	0.8435	0.9346	0.9495	5.802	2.925	5.301	63	71	46	20
96	***FLT3*** ^{ITD}	0.8821	0.8535	0.8941	0.887	0.7228	0.6391	0.7268	0.7449	4.509	2.015	3.801	65	69	37	23
41	***FLT3*** ^{TKD}	0.6756	0.592	0.6362	0.6389	0	0	0	0.1739	1.479	0.800	1.328	32	21	24	8
41	***FLT3*** ^{ITD} **/ *NPM1*** ^{wt}	0.8147	0.6834	0.8194	0.7876	0.3955	0	0.3438	0.5292	1.547	1.238	1.707	61	36	41	19
50	***FLT3* / *NPM1*** ^{mutant}	0.9467	0.865	0.9504	0.9522	0.6722	0.4679	0.6236	0.6945	3.336	1.392	2.762	57	60	42	22
55	***FLT3*** ^{ITD} **/ *NPM1*** ^{mutant}	0.9441	0.9378	0.9378	0.9513	0.6789	0.6156	0.6591	0.6859	3.948	1.740	3.335	43	53	28	12
35	***NRAS***	0.7617	0.7617	0.7593	0.7727	0.08889	0	0.04444	0.2405	2.075	0.354	1.196	43	37	30	8
22	**3q**	0.6611	0.7415	0.6967	0.7291	0.1143	0.05714	0.181	0.3032	0.911	0.749	0.912	26	21	21	8
35	**7q**	0.7947	0.7333	0.799	0.7998	0.5626	0.18	0.516	0.6897	1.287	1.205	1.787	28	48	20	6
31	**11q23**	0.8702	0.766	0.846	0.8729	0.6489	0.52	0.6089	0.7067	2.294	1.696	2.460	34	28	27	6
